# Differences in Faecal Microbiome Taxonomy, Diversity and Functional Potential in a Bovine Cohort Experimentally Challenged with *Mycobacterium avium* subsp. *paratuberculosis* (MAP)

**DOI:** 10.3390/ani13101652

**Published:** 2023-05-16

**Authors:** Chloe Matthews, Aaron M. Walsh, Stephen V. Gordon, Bryan Markey, Paul D. Cotter, Jim O' Mahony

**Affiliations:** 1 Department of Biological Sciences, Munster Technological University, Bishopstown, T12 P928 Cork, Ireland; 2 Teagasc Food Research Centre, Moorepark, P61 C996 Fermoy, Irelandpaul.cotter@teagasc.ie (P.D.C.); 3 School of Veterinary Medicine, University College Dublin, D04 V1W8 Dublin, Ireland; 4 APC Microbiome Ireland, University College Cork, T12 R229 Cork, Ireland

**Keywords:** Johne’s disease, MAP, microbiome, experimental model, immune function

## Abstract

**Simple Summary:**

Johne’s disease is a global economic burden. The disease contributes to reduced milk yield, reduced fertility, higher rates of susceptibility to other diseases and eventual death. Unhealthy animals are less efficient, producing higher kg of greenhouse gases per kg of output (milk and meat). Therefore, prognosis and diagnosis are important in terms of animal welfare and future climate change policies by reducing the impact of livestock on climate change. In the past decade, our knowledge around the microbiome in health and disease has increased. Next generation sequencing technologies have provided a new understanding of the interactions between the host microbiome and pathogens. The microbiome is a target for treatment, prevention of disease and a source of novel biomarkers of disease risk. The potential to improve animal health is highly dependent on our ever growing knowledge of the gut microbiome and their interactions with the host. With the aim to gain knowledge in the early months of exposure to MAP in calves, we investigated the dynamics of the gut microbiome (using faecal sample) of calves 3, 6 and 9 months post inoculation with MAP relative to unexposed controls.

**Abstract:**

Mycobacterium avium subspecies paratuberculosis (MAP) is the causative agent of Johne’s disease in ruminants, a chronic enteritis which results in emaciation and eventual loss of the animal. Recent advances in metagenomics have allowed a more in-depth study of complex microbiomes, including that of gastrointestinal tracts, and have the potential to provide insights into consequences of the exposure of an animal to MAP or other pathogens. This study aimed to investigate taxonomic diversity and compositional changes of the faecal microbiome of cattle experimentally challenged with MAP compared to an unexposed control group. Faecal swab samples were collected from a total of 55 animals [exposed group (*n* = 35) and a control group (*n* = 20)], across three time points (months 3, 6 and 9 post-inoculation). The composition and functional potential of the faecal microbiota differed across time and between the groups (*p* < 0.05), with the primary differences, from both a taxonomic and functional perspective, occurring at 3 months post inoculation. These included significant differences in the relative abundance of the genera Methanobrevibacter and Bifidobacterium and also of 11 other species (4 at a higher relative abundance in the exposed group and 7 at a higher relative abundance in the control group). Correlations were made between microbiome data and immunopathology measurements and it was noted that changes in the microbial composition correlated with miRNA-155, miR-146b and IFN-ɣ. In summary, this study illustrates the impact of exposure to MAP on the ruminant faecal microbiome with a number of species that may have relevance in veterinary medicine for tracking exposure to MAP.

## 1. Introduction

*Mycobacterium avium* subspecies *paratuberculosis* (MAP) is the causative agent of Johne’s disease, a chronic enteritis, principally affecting the distal ileum. The pathogen induces thickening and corrugation of the intestinal wall, causing emaciation and eventual animal loss. MAP is found worldwide, and its spread is of major concern. In particular, globalisation has led to an increase in the movement of goods and commodities, including livestock and livestock products [1], with associated risk of the spread of infectious diseases in animals. Therefore, rapid diagnosis using minimal quantities of easily accessible biological samples may pave the way for protection of animal production systems in herds (at a local level) and countries with a disease-free status. While direct detection of pathogens is ideal in situations where this is challenging, indirect markers of infection or exposure merit consideration. In this regard identifying changes in the gastrointestinal (GIT) microbiome that occur in response to pathogen infection or exposure to a pathogen has the potential to be of value. This reflects the fact that next generation sequencing has transformed microbiological research by enabling high throughput metagenomic analysis of complex microbial communities from a number of different environments and hosts [2,3,4,5]. Metagenomic analysis has already revealed changes in the microbiome associated with exposure to pathogens in cattle [6,7], pigs [8,9], mice [10], and humans [11]. Such an approach, if developed, could be particularly valuable for the detection of difficult isolates, slow-growing pathogens such as MAP. These microorganisms require specialist culture media and laboratory protocols, while growth can take place from approximately 8–12 weeks for Type C strains and longer for Type S strains, while in some instances culture is not always successful.

The microbiome in early life plays an important role in health across mammalian species. In calves, the development of the microbiome is influenced by a number of different factors including diet (colostrum, milk replacer or calf starter), exposure to the dam, antibiotic use, the age of the animal and the sampling location [12]. The establishment of an optimal microbiome has been positively associated with calf health and growth, the prevention of neonatal diarrhoea [13] and pneumonia [14] as well as a rapid increase in weight [12]. Microbial colonisation also contributes to the development of the intestinal epithelial, mucosal layer and lymphoid structures [15]. In contrast, exposure to pathogens early in life may be detrimental to the host, impacting on short- and/or long-term health and productivity [12]. Although calves may be infected with MAP through intra-uterine transmission in clinically affected cows, it is more common via the faecal-oral route when they are exposed to faecal contaminated teats, pasture, water, supplements, bedding, milk or colostrum [16].

There have previously been a small number of 16S rRNA-based analyses of the faecal microbiome of animals naturally infected with MAP relative to controls. In one instance, an over-representation of the families Planococcaceae and Paraprevotellaceae and an under-representation of the genera *Faecalibacterium* and *Akkermansia* were noted in infected cattle [17]. Enrichment of lysine and histidine metabolism pathways and an underrepresentation of glutathione metabolism and leucine and isoleucine degradation pathways within the ileal mucosa-associated microbiome of MAP-infected cattle was also shown using the predictive software, PICRUSt (https://github.com/picrust/picrust, accessed on 25 February 2023). In a separate study, an increase in the genus *Psychrobacter* and decrease in the genera *Oscillospira*, *Ruminococcus* and *Bifidobacterium* was reported in cows infected with MAP [18]. Fecteau et al., 2016 [19] showed differences in the faecal microbiome between MAP-positive and MAP negative cattle using 16S rRNA sequencing technologies, with the microbiome of MAP-positive animals having a higher abundance of Actinobacteria in comparison to controls.

In addition to studies of naturally infected animals, there have been a number of studies whereby cattle have been experimentally challenged with MAP [20,21]. Experimental challenge models have the potential to provide useful information including host immune response following exposure and corresponding interactions with the normal gut microbiome. Such studies were carried out by Rankin and colleagues when MAP was known as *Mycobacterium johnei*. The group used large, single oral doses of MAP in their studies to investigate the portal of entry [22]. Other experiments, such as those conducted by [23], used smaller doses but inoculated weekly. More recently, a long-term MAP experimental study was conducted in Holstein Friesian cattle over a 33 month period [24,25]. It was found that there was significant differential expression of miR-155 in animals exposed to MAP. MiRNAs have been used to detect changes in animals associated with exposure to pathogens, with several studies using them as biomarkers for disease. Differential miRNA profiles in local anatomical sites may facilitate better understanding of the immunopathology of MAP infection [25].

The main aim of this study is to explore the taxonomy and metabolic activity of the microbiome of calves experimentally challenged with MAP relative to controls to determine if MAP exposure impacts the associated faecal microbiome. Diversity measures between exposed and control groups will be examined to determine if exposure to MAP influences the diversity profile of the microbiome. Associations between gut microbiome composition and immunomodulatory measures including miRNAs miR-155 and miR-146b, ELISA sero-positivity and IFN-ɣ will also be determined.

## 2. Materials and Methods

### 2.1. Animals and Experimental Design

This study employed samples collected from a long-term MAP experimental challenge. Specifically, rectal faecal swabs were collected from a group of animals that were exposed to MAP (*n* = 35) and a control group (*n* = 20), across three time points (months 3, 6 and 9 post-inoculation) [24].

Detailed information relating to animal selection criteria and husbandry can be found in the thesis of Britton 2017 [25]. Briefly, 55 Holstein-Friesian calves were sourced from two Autumn calving herds (herd A and herd B). Both farms had MAP seropositivity rates of ≤10% and neither farm had a history of Johne’s disease. All dams were MAP faecal culture negative and seronegative. In terms of diet in early life, calves received 2–4 L of colostrum from their own dam shortly after birth and were then removed from the mothers. Calves were housed indoors in small groups and received Gain^®^ Easi-Mix calf milk replacer (Gain Feeds, Portlaoise, Ireland) until weaning at approximately 3 months of age. Animals were then maintained on an ad libitum silage-based diet. Thirty five calves aged between 3 and 6 weeks were orally inoculated on two consecutive days with 10 mL (3.8 × 10^9^ CFU) of MAP strain CIT003. A control group of 20 calves received a placebo. The animals were housed indoors in group housing on a semi-slatted unit with access to a solid-floor, straw-bedded area, for the duration of the experiment with the groups housed separately (approximately 0.5 km apart). Blood, faecal and faecal rectal swab samples were collected from all animals throughout the trial at regular intervals (premmune and 2, 3, 6, 10, 12, 16, 20, 24, 28, 31 and 33 months post inoculation and premmune and 3, 6, 9, 12, 16, 20, 24, 28, 31 and 33 months post inoculation, respectively) in order to determine MAP infection status. Cell-mediated immunity was measured using an IFN-ɣ assay (Bovigam^®^) test and serum anti-MAP antibodies were measured using a commercially available kit (Idexx Johne’s Identification and Verification ELISA, IDEXX Laboratories, Inc. One IDEXX Drive, Westbrook, ME, USA). Faecal samples were cultured for 42 days using the TREK ESP para-JEM system (Thermo Fisher Scientific, Waltham, MA, USA). Briefly, faecal samples were mixed thoroughly before 2 g was suspended in 35 mL sterile distilled water. The samples were then vortexed and allowed to stand for 30 min. About 5 mL of the resuspended faecal material was added to 25 mL 0.9% hexadecylpyridinium chloride in 50% brain heart infusion broth and was incubated overnight at 37 °C. Replicates, taken from the same samples were transported on foam swabs, before being frozen at −80 °C until DNA extraction. Storage of swabs at −80 °C allowed for faecal rectal samples to be processed at a later date for DNA extraction and microbiome analysis. Confirmatory MAP specific histological changes (including tissue from distal ileum and ileocecal lymph nodes) were not detected during the 33 month trial, and it is unknown whether any of the challenged animals would have progressed to faecal shedding of MAP and disease. These tissue samples were collected at post-mortem examination at months 12, 24 and 33 post inoculation. The number of animals euthanized at each timepoint is as follows; 8 exposed and 5 controls were euthanized at timepoints 12 and 24, while 19 exposed and 9 controls were euthanized at month 33 (1 animal from the control group was euthanized at month 16 due to issues unrelated to the trial). ELISA seropositivity and IFN-ɣ was also be determined via commercial Bovigam^®^ (Prionics AG, Schlieren, Switzerland).

### 2.2. DNA Extractions and Library Preparations

DNA extractions were carried out on 165 faecal swabs from months 3, 6 and 9 post inoculation using the Qiagen PowerSoil Pro kit (Qiagen Inc., Germantown, MD, USA). One sample (from the control group) was lost during DNA extraction (*n* = 164). The foam tip of each faecal swab was cut under sterile conditions into initial bead beating tubes supplied in the extraction kit. Extractions were performed as per protocol with an additional heat step, post bead beating, at 70 °C for 15 min which was adapted from [26]. The heat step was performed in bead-beating microfuge tubes as per [26]. Extracted DNA was quantified using the Qubit High Sensitivity DNA assay (Biosciences, Dublin, Ireland). Whole-metagenome shotgun libraries were prepared using the Illumina Nextera XT DNA Library Preparation Guide except that the tagmentation time was increased from 5 to 7 min. Samples were sequenced on the Illumina NextSeq platform at the Teagasc sequencing facility (Teagasc, Food Research Centre, Moorepark, Co. Cork, Ireland), using the high output reagent cartridge V2 300 cycle kit, following standard Illumina sequencing protocols.

### 2.3. Bioinformatics Analysis of Exposed vs. Control Animals across Time

Raw whole-genome shotgun sequencing reads were filtered on the basis of quality and quantity using the KneadData package using default parameters (http://huttenhower.sph.harvard.edu/kneaddata, accessed on 25 February 2023). Bovine genome reads (*Bos taurus*) were filtered and excluded from further analysis. Compositional assignment was carried out using MetaPhlAn2 using default settings [27]. The functional potential of the microbiome was determined using SUPER-FOCUS [28].

### 2.4. Statistical Analysis Comparing Exposed to Controls and across Time, and Correlating Immunopathology with Taxonomic Output

Statistical analysis was carried out in R studio, R version 3.5.3 (11 March 2019) (RStudio, Inc. PBC, Boston, MA, USA). Analysis was carried out to compare the microbiome of animals in exposed versus control groups and across time (be-tween months 3, 6 and 9). Data were checked for normality using the Shapiro-Wilks normality test in R. Each test for each dataset being used returned a *p*-value of <0.0001, indicating the data were non-normal. Alpha diversity analysis was carried out using the “vegan” package using Shannon, Simpson and Observed Species indices, in addition to multidimensional scaling (MDS). The Wilcoxon rank sum test was used to determine statistical differences in alpha diversity between groups. Permutational analysis of variance (PERMANOVA, was performed using the “adonis” function from vegan (version 2.5-6). Results from significance testing using adonis were confirmed using multivariate analysis using the betadisper function from the “vegan” package. This measures the variance of a group of samples by calculating the average distance of group members to the group centroid (https://www.rdocumentation.org/packages/vegan, accessed on 25 February 2023). PPD-j induced IFN-ɣ and sero-positive results were correlated (Spearman Rank correlation using the “corrplot” package) with microbial abundances. The same approach was applied to identify correlations with the ex-pression of microRNAs; miR-16, miR-21, miR-29a, miR-146b, miR-155 and miR-223, and cell-mediated immunity, measured using an IFN-ɣ assay using purified protein derivative of MAP (PPD-J) was carried out by [25]. For this test, 22.5 ug/mL MAP PPDj (kindly provided by Dr. Douwe Bakker, Central Veterinary Institute, Lelystad, The Netherlands) was used.

The linear discriminant analysis (LDA) effect size was determined using LEfSe [27]. LEfSe uses the two-tailed non-parametric Kruskal–Wallis test to examine the significance of differences of species and functional potential in the two groups. The groups were analysed separately across time. A set of pair-wise tests among the two groups was performed using the Wilcoxon test. Finally, LDA was performed to estimate the effect size of each differently abundant species. The microbiome of each of the groups was thought to be significantly different if their differences had a *p*-value < 0.05 and an LDA score (log10) > 2. Data visualisation was performed using the “ggplot2” package in R.

## 3. Results

### 3.1. The Taxonomic Profile of Calves Exposed to MAP Differs from Controls

**Figure 1:**
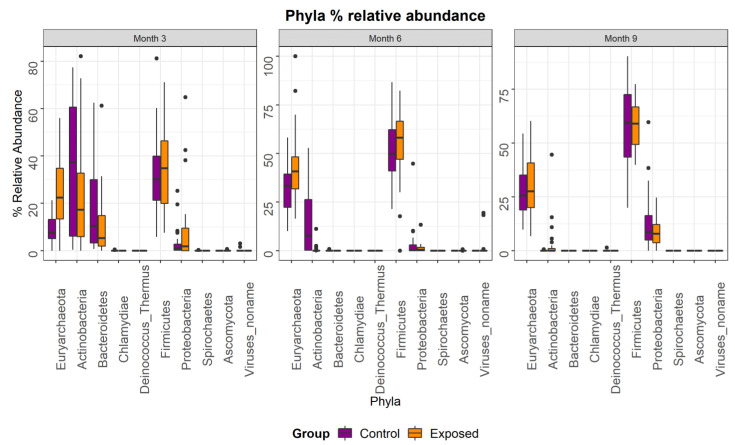
Phylum level analysis revealed an increase in the relative abundance of members of Euryarchaeaota and Firmicutes in the exposed group with a decreased relative abundance of Actinobacteria and Bacteriodetes in the exposed group, with the greatest differences observed in month 3.

**Figure 2:**
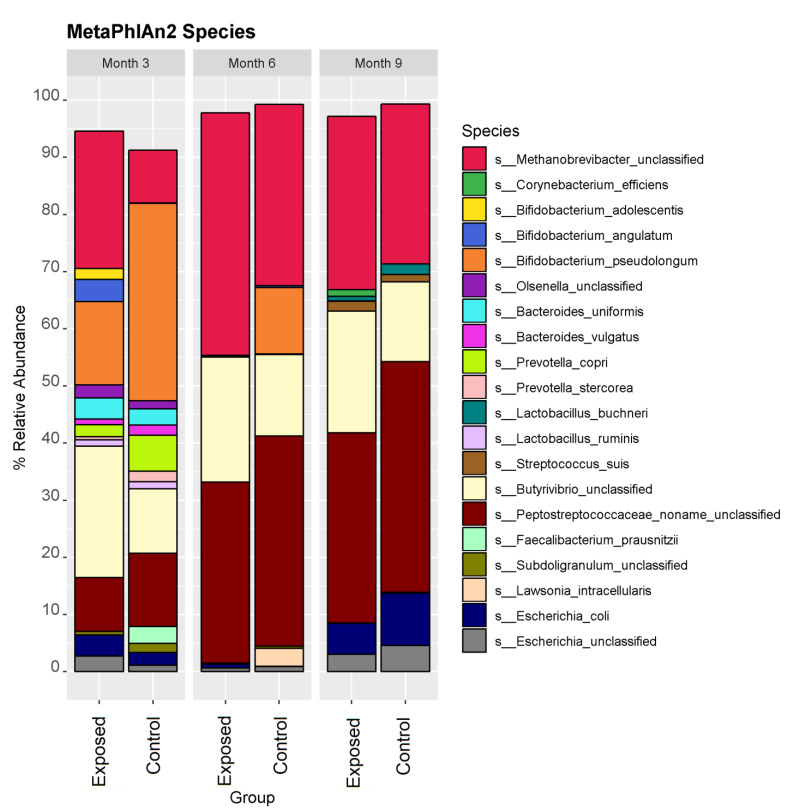
The microbiome was dominated by a combination of four species; Methanobrevibacter unclassified, Bifidobacterium pseudolongum, Butyrivibrio unclassified and Peptostreptococcaceae unclassified.

**Figure 3:**
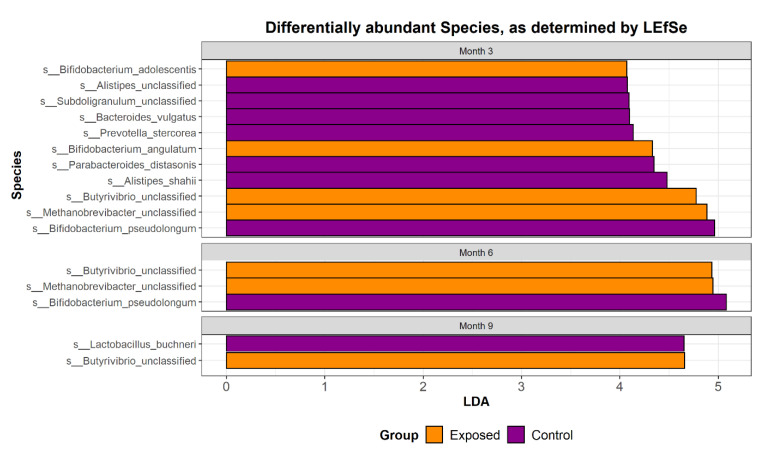
Discriminatory species were identified using linear discriminant analysis (LDA) effect size (LEfSE), which allows for the identification of species which explain differences between groups. Abundances of Bifidobacterium species decreased over time, particularly *B. pseudolongum*, *B. angulatum* and *B. adolescentis*.

Metagenomic sequencing was used to investigate how exposure to MAP at 3–6 weeks of age impacts the composition and functional potential of the faecal microbiome of calves across three time points post inoculation. In total, the study consisted of 164 samples from 35 exposed and 20 control animals across three time-points (months 3, 6 and 9 post inoculation). Rectal faecal samples and tissue samples remained culture negative throughout the 33 month challenge. However, taxonomic profiling using MetaPhlAn2 revealed a significant difference (*p* < 0.05) between groups across time with respect to diversity and composition. Phylum level analysis revealed an increase in the relative abundance of members of Euryarchaeaota and Firmicutes in the exposed group with a decreased relative abundance of Actinobacteria and Bacteriodetes in the exposed group (Figure 1). The relative abundance of the top 25 most dominant species in each individual animal was examined. Overall, the faecal microbiome, from both the exposed and control groups, was dominated by a combination of four species; *Methanobrevibacter unclassified*, *Bifidobacterium pseudolongum*, *Butyrivib-rio unclassified*, and *Peptostreptococcaceae unclassified* (Figure 2). Discriminatory species were identified using linear discriminant analysis (LDA) effect size (LEfSE), which allows for the identification of species or pathways which explain differences between groups (Figure 3). Abundances of Bifidobacterium species decreased over time, particularly *B. pseudolongum*, *B. angulatum* and *B. adolescentis* (Figure 3). LEfSE also showed a number of species that significantly differed (*p*-value < 0.05) in relative abundance between the groups (Figure 3). Supporting data can be found in Supplementary Table S1. These included an unclassified Methanobrevibacter species that was present at significantly greater relative abundance in the exposed group at months 3 and 6 and an unclassified species of Butyrivibrio that was significantly more abundant in the exposed group across all time points. *B. angulatum* and *B. adolescentis* were significantly more abundant in the exposed group at month 3, whereas *B. pseudo-longum* was significantly more abundant in the control group at months 3 and 6. Overall, month 3 showed the greatest number (11) of significant differences, with 7 at higher relative abundance in the control group and 4 at higher relative abundance in the exposed group. Other taxa that were more abundant in the control group at month 3 were *Alistipes shahii*, *Parabacteriodes distasonis*, *Prevotella stercorcea*, *Bacteroides vulgatus* and *Subdoligranulum unclassified*. It is important to note that MAP sequences were not detected at any time point.

### 3.2. Differences in Faecal Microbiome Alpha and Beta Diversity between MAP Exposed and Control Animals

**Figure 4:**
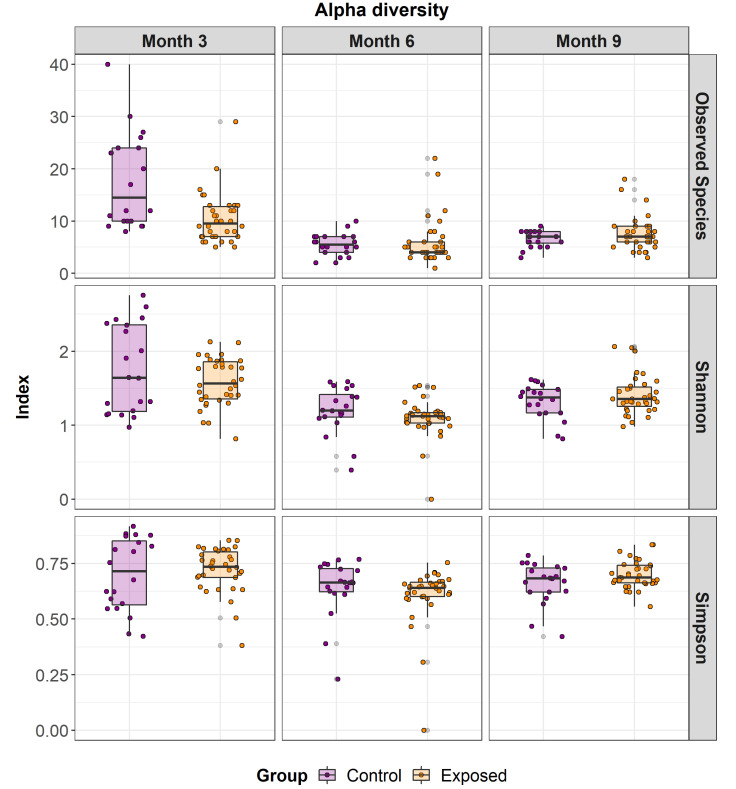
Diversity of the microbiome of both the exposed and control animals across time. Alpha diversity within subjects using Shannon, Simpson and Observed species measures of species-level output from MetaPhlAn2. The control group shows a higher within animal diversity; however, this was non-significant.

Alpha diversity analysis showed differences over time; alpha diversity decreased over time specifically between month 3 and month 9. More specifically, differences in the species-level alpha diversity (observed species, Shannon and Simpson index) of the faecal microbial communities within the groups (exposed versus control) were determined for all samples (Figure 4).

**Figure 5:**
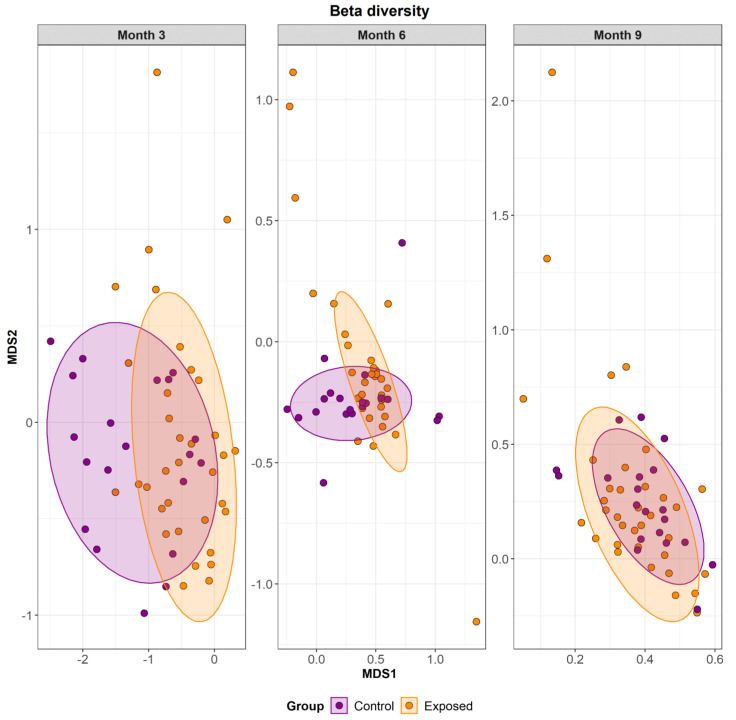
Bray-Curtis beta diversity among animals in both the exposed and control group, across time. Month 3 shows the most variation between the groups. The microbiome becomes more stable as animals age or potentially as a result of MAP leaving the system as can be observed at the month 6 and 9 time points. MDS1 and MDS2 represent the two dimensional space in which the points are arranged.

With respect to beta diversity, multidimensional scaling was used to visualise the level of similarity between samples, as calculated using the Bray-Curtis dissimilarity metric. PERMANOVA revealed significant differences between groups (*p* < 0.001) and between sero-positive and sero-negative animals (*p* < 0.05). Time-related changes can be observed in the Bray-Curtis index values as overall community structure changed across time (Supplementary Table S2), and progressed towards a more similar microbiome (Figure 5). Microbiomes from samples collected at month 3 had a significantly higher variability than samples collected at months 6 and 9. Betadisper analysis confirmed significant differences between both the exposed and control animals and in month 3 vs. month 6 and month 3 vs. month 9. Betadisper analysis also confirmed that exposed animals were less similar to each other whereas control animals showed more similarity (*p* < 0.01) (Supplementary Table S2). Differences related to herd of origin can be found in (Supplementary Figure S2).

### 3.3. The Functional Potential of the Microbiome of Animals Exposed to MAP Differs from That of Controls

**Figure 6:**
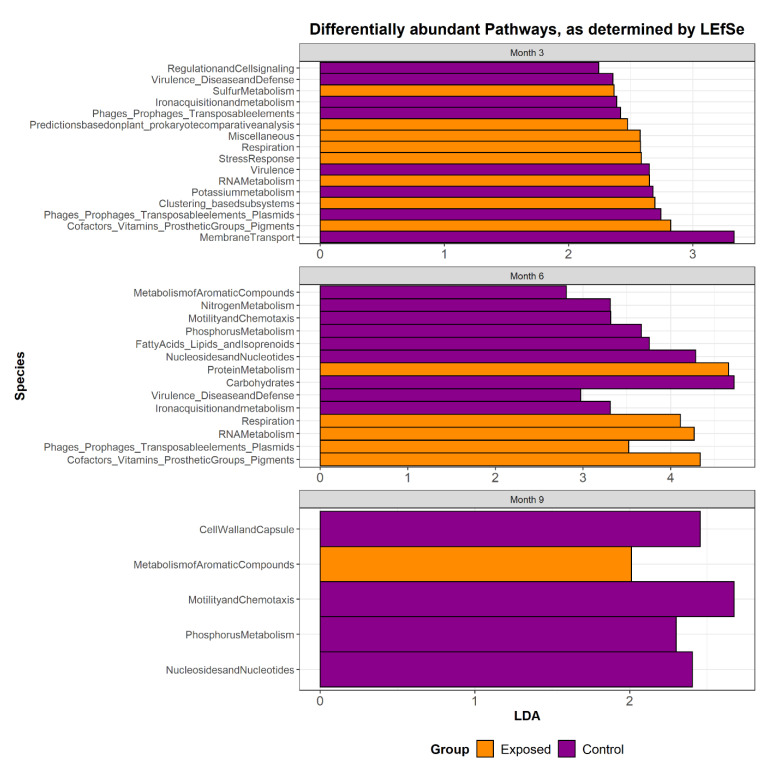
Functional potential of the microbiome at level 1 subsystem from the SUPER-FOCUS output. SUPER-FOCUS uses the SEED database, which is a subsystem database, assigning function at four different levels. Subsystems are divided into hierarchies. Number s on the x axis refers to the linear discriminant analysis (LDA) score. The linear discriminant analysis (LDA) effect size was determined using LEfSe. LEfSe uses the two-tailed non-parametric Kruskal–Wallis test to examine the significance of differences of species and functional potential in the two groups. A set of pairwise tests among the two groups was performed using the Wilcoxon test. LDA was performed to estimate the effect size of each differently abundant species. The functional potential of each of the groups was thought to be significantly different if their differences had a *p*-value < 0.05 and an LDA score (log10) > 2.

Analysis of the functional potential of the faecal microbiomes revealed that genes related to sulphur metabolism, respiration, stress response, RNA metabolism, clustering based subsystems, co-factors and vitamins were significantly (*p* < 0.05) enriched in the exposed group 3 months’ post-challenge, relative to month 3 controls. Genes associated with membrane transport, potassium metabolism, virulence, iron acquisition and metabolism, regulation and cell signalling, and phages, prophages and transposable elements were more abundant in the control group. A similar number of significantly different pathways was noted at month 6 but a smaller number of differences was evident at month 9 (Figure 6). A more in-depth analysis of functional data established that genes associated with a number of hydrogenase pathways, including formate hydrogenase, were present at significantly higher relative abundance in the exposed group overall. Maltose and maltodextrin utilisation, glycogen metabolism, glycolysis and gluconeogenesis and genes associated with the resistance of fluoroquinolones were also more abundant in the exposed group. Genes associated with Ton and Tol transport systems (a major group of pattern recognition receptors), cellulosomes, mannose metabolism, histidine degradation and ammonia assimilation pathways were more abundant in the control group (Supplementary Figure S1, Supplementary Tables S3 and S4).

### 3.4. Correlations between the Gut Microbiome and Immunopathology Measurements Reveals Important Associations between the Microbiome and Host Immune Response

In total, 12 animals were seropositive at one or more timepoints across the 33 month trial from month 16–33. Spearman Rank Correlations were used to examine the correlations between faecal microbiome species and seropositive animals. A sero-positive result reflects an instance where an animal had one ELISA positive result during the duration of the trial. Seven taxa were found to have significantly positive (*p* < 0.05) correlations with sero-positive results (ρ ranged between 0.16 and 0.22) (Table 1). *Enterococcus hirae* appears in 4 animals from the exposed group that had sero-positive results specifically at 3 months post inoculation in comparison to animals from the same timepoint in the control group. This species did not appear at other timepoints. ELISA results can be found in Supplementary Table S5.

Circulating miRNAs have been shown to have significant potential as biomarkers for a range of human diseases. Notably, miR-155, an important regulator of gene expression that has the ability to control infection and inflammation in the gut, was significantly decreased in the exposed group in comparison to the control group [25]. The further analysis of the newly generated faecal microbiome data revealed that the relative abundances of *C. efficiens* and *B. adolescentis* were negatively correlated, and *B. pseudolongum,* unclassified *Alistipes* and *Desulfovibrio piger* were positively correlated, with the calibrated normalised relative quantity of miR-155 (Table 2). The only other significant correlation was a positive correlation between *B. adolescentis* and expression of miR-146b, an miRNA which, when overexpressed activates and upregulates the NF-κB pathway, thereby inhibiting autophagy, improving intestinal epithelial function and reducing intestinal inflammation [28].

Finally, PPD-j induced IFN-gamma levels from 3 months post inoculation positively correlated with Bacteroides thetaiotaomicron, Bacteroidales bacterium_ph8, Dorea formicigenerans, Faecalibacterium prausnitzii and Escherichia unclassified (Table 3). PPDj results for month 3 post inoculation can be found in Supplementary Table S6.

## 4. Discussion

Several studies have shown the host microbiome can change in response to both pathogen exposure and infection [19,29,30]. However, relatively little is known about how host microbiomes may respond to MAP exposure. Our study identifies changes in the microbiome of calves inoculated with a high dose of MAP relative to controls. These differences are most apparent at 3 months’ post inoculation. We also identify associations between gut microbiome composition and immunomodulatory measures including miRNAs miR-155 and miR-146b, ELISA sero-positivity and IFN-ɣ. Further differences were highlighted through diversity analysis between the exposed and control groups.

There have been some previous studies of the GIT microbiomes of animals exposed to MAP. Authors of [19] showed differences in the faecal microbiome between MAP-positive and MAP negative cattle using 16S rRNA amplicon-based sequencing, with the microbiome of MAP-positive animals having a higher abundance of Actinobacteria in comparison to controls. Our results also show a higher abundance of members of the phylum Actinobacteria, including *Bifidobacterium* spp., in the MAP exposed group. In addition, we also identified an increase in the relative abundance of members of Euryarchaeaota and Firmicutes, with a decreased relative abundance of Actinobacteria and Bacteriodetes, in the MAP-exposed group. Generally, differences were most evident 3 months post inoculation. Our analysis highlighted a number of species that may have relevance with respect to identifying MAP exposure, including a higher abundance of *Methanobrevibacter* in challenged animals. Archaea have not yet been reported in the pathogenesis of MAP and may warrant further investigation. The higher abundance of methane producing archaea in the exposed group may result in higher methane production in the lower gastrointestinal tract. Non-invasive methods to detect this include the measurement of methane emissions through open-circuit respiration chambers may be of value from the perspective of the detection of methane emissions in flatus and faeces. Given that other methane emission units are designed for eructed methane (to estimate methane from rumen fermentation), the open circuit chamber may be the most appropriate method given that methane under examination is methane generated in the lower gastrointestinal tract.

Furthermore, a number of *Bifidobacterium*, including *B. angulatum*, were present at higher relative abundance in the MAP-exposed group. This species is considered a specialised member of the *Bifidobacterium* genus and had been thought to be exclusively associated with animal faeces [31,32]. However, the species has also been reported in a number of Crohn’s disease and ulcerative colitis studies [33]. Notably, [33] examined antibody-mediated immune responsiveness to the cell surface of mucosal bacteria for IgG and IgA serum antibodies and noted that *B. angulatum* IgG responses were significantly higher in the ulcerative colitis cohort. The other *Bifidobacterium* species enriched in the exposed group was *B. adolescentis*. This species is known to produce lactate and a small quantity of acetate that may drive butyrate formation in other bacteria. Other taxa enriched among exposed animals included a variety of representatives of the genus *Butyrivibrio*. *Butyrivibrio* are butyrate producers with significant importance as they promote T-cell differentiation that can ultimately suppress pro-inflammatory responses [34]. Butyrate supplementation of the diet of calves has also been shown to have a beneficial effect in terms of growth and performance [35], potentially due to the regulation of the immune system and maintenance of the epithelial barrier. From the other extreme, *B. pseudolongum* was found at a lower relative abundance in the exposed group. This may be a result of competition for an ecological niche with other *Bifidobacterium* species. *Prevotella stercorea* was also present at lower relative abundance in the exposed group in month 3 in comparison to the exposed group. According to authors of [36], this species has a weak negative correlation with *E. coli* O157:H7 prevalence and enumeration in the faecal microbiome of cattle. Although MAP sequences were not detected, alteration in the gut microbiome suggests exposure to MAP. MAP was inoculated at a high dose but may have been passed from the animal in the days and weeks following inoculation. The changes found between groups at month 3 and weaning at months 6 and 9 may be a result of MAP passing through the GIT. This in itself is interesting as it indicates that if an animal is resistant to infection, it may take up to 6 months for the microbiome to fully recover from exposure. To fully identify if this was the reason, samples from months 1 and 2 post-inoculation should have been taken. These samples were not available for this study but should be noted for future investigations. It is also recommended to take samples in the days that follow inoculation to identify how long it takes for MAP to be cleared for the GIT when infection does not occur. The presence of potentially beneficial microbes including species of Bifidobacterium may also indicate that these microbes are aiding in the recovery of the microbiome. However, the influence of the environment on the microbiome cannot be ignored. *Enterococcus hirea* was detected at month 3 but was not detected in months 6 or 9. This may be a result of housing conditions and not experimental conditions.

Changes across time are highly likely to be a result of age and dietary modifications. Gradual weaning of calves from milk or commercial milk replacer typically takes place after the 12th week of life, where the animals are gradually introduced to calf starter feed which usually consist of concentrate (18–20% crude protein and less than 7% fibre) and forage. Overall, the community was highly variable with increasing alpha diversity but decreasing inter-animal variation (beta diversity) as the animals aged, a pattern that is consistent with the previous studies of the calf microbiome [37]. As discussed above, factors which may influence the microbiome during these timepoints may include dietary changes, changes in the environment and maturing of the intestinal microbiome. Alpha diversity was lower in the exposed group in comparison to the control group. Low diversity is a common feature of a disturbed microbiome, which in this case was due to the exposure to MAP.

The functional potential of the faecal microbiome was examined showing significant differences in pathways related to sulphur metabolism, respiration, stress response, RNA metabolism, clustering based subsystems, co-factors and vitamins which were enriched in the MAP exposed relative to the control group at month 3 post exposure. Genes associated with membrane transport, potassium metabolism, virulence, iron acquisition and metabolism, regulation and cell signalling, and phages, prophages and transposable elements were more abundant in the control group. Virulence factors found in MAP may have downregulated these pathways found in the microbiome, contributing to the higher relative abundance found in the control group. Bacterial derived hydrogen may contribute to the proliferation of methanogens in months 3 and 6, which may be a consequence of MAP exposure. Effect of methanogens is interpreted as a shift in the flow of electrons away from the formation of electron sink products lactate and ethanol to methane via hydrogen, favouring an increase in acetate which is in turn converted to methane and carbon dioxide. MAP needs to convert superoxide to hydrogen peroxide. Therefore, hydrogen peroxide may be important in methane production, as there is an increase in available hydrogen. A higher abundance of genes associated with hydrogenase pathways in the exposed group may contribute to the higher number of methanogens seen in the exposed group in the early days of exposure. Hydrogen peroxide is thought to have a positive effect on methane production and is used in a number of industrial systems to increase methane production.

Pathways involving sulphur metabolism pathways within the GIT microbiome of humans can have important roles; however, less is known about their role in cattle. When it is not assimilated, the end product of the anaerobic microbial degradation of sulphur-compounds is predominantly hydrogen sulphide (H_2_S). Gastrointestinal H_2_S is a neuromodulator and plays a critical role in controlling physiological responses such as motility and epithelial cell health. It has also been suggested that H_2_S has a potential pathogenic role, such as with respect to inflammatory bowel disease in humans. A number of bacterial taxa have been associated with sulphur metabolism, including *M. tuberculosis* in the GIT. Genes associated with stress response were enriched in the microbiome of the exposed group, which may reflect a stressful environment arising as a consequence of MAP exposure.

The microbiome has long been implicated in the activation or suppression of the immune system in response to pathogens. Correlations with IFN-ɣ and seropositivity were made for a number of species with immunogenic properties. Commensal microbes are important modulators of host physiology, metabolism and immunity. Immunomodulatory effects of commensal microbes have been noted; for example, studies have examined *Enterococcus hirae* as a therapeutic immunomodulatory microbe, with a role in memory Th1 immune response. Subclinical Johne’s disease is characterised by a Th1 response effective at controlling and limiting the spread of intracellular infections [38]. Although found in only 2 samples at a mean abundance of 0.14, it was noted that *Erysipelotrichaceae bacterium* 21_3 was significantly positively correlated with sero-positive animals. Members of this family are thought to be highly immunogenic [39].

The potential of microbiome-activated immunity was explored through identifying correlations between immunomodulatory measures and relative abundances of microorganisms present in the gut of exposed and control animals. Weak correlations were made with a number of taxa, namely *B. thetaiotaomicron*, *C. efficiens*, *Butyrivibrio unclassified* and *Bifidobacterium* spp., some of which have been discussed in detail above, were noted. These were found in only a small number of animals but may warrant further investigation in studies where animals progress to an infected state. Of the others, *B. thetaiotaomicron* is notable as this species encodes a number of glycoside hydrolases and polysaccharide lyases, degrading complex polysaccharides to monosaccharides that can be readily used by non-glycophagic species, overall, enriching the availability of nutrients in the gut [40,41] and inhibiting the effects of IFN-ɣ on epithelial function [42]. As miRNAs are also a promising target for Johne’s disease prognosis, correlating miRNAs with the microbiome may provide a robust method for novel prognostics. New targets are regularly under investigation. Recently, authors of [43] found two miRNAs of interest associated with immune response to MAP infection.

There are a number of ways through which the investigations described in this study can be further advanced. Although, faecal samples in this study were collected via rectal swabs, collection of samples through ileal cannulation can provide a better indication of the regional microbiome associated with MAP exposure. Although this type of study could better inform underlying immunopathology associated with MAP exposure, as an invasive procedure it would require robust justification on welfare grounds. Ingestible sensors are among a wave of new technologies that may improve the way in which we sample the microbiome. At present, faecal samples provide the best proxy for GIT microbiome representation. In light of this, a more rigorous sampling period in the hours and days post-inoculation in experimental models may be needed to eliminate the possibility of excretion of the microorganism during this timeframe.

Despite its limitations, this study provides new insights into the molecular mechanisms underpinning MAP exposure in calves, particularly at 3 months post inoculation. With this being said, results should be used cautiously. Extensive work on naturally infected animals would be needed to further verify the results achieved in this study. This study adds to the growing amount of data relating to the impact of exposure to a pathogen on the microbiome. We show that the microbiome changes over time in response to exposure and have identified a number of potential biomarkers that merit further investigation. In parallel, the merits of carrying out immunological and pathological analyses in conjunction with microbiome studies are highlighted.

## Figures and Tables

**Table 1:** Correlation coefficient and *p*-values of species associated with an ELISA seropositive result using Spearman rank correlation.

Species	*p*-Value	Correlation Co-Efficient
*Methanobrevibacter ruminantium*	0.039	0.162
*Corynebacterium efficiens*	0.007	0.211
*Bacteroides thetaiotaomicron*	0.045	0.157
*Enterococcus hirae*	0.007	0.209
*Butyrivibrio unclassified*	0.005	0.220
*Erysipelotrichaceae bacterium* 21_3	0.006	0.213
*Penicillium chrysogenum*	0.006	0.213

**Table 2:** Correlation coefficient and *p*-values of species associated with miR-155 expression using Spearman rank correlation.

Species	*p*-Value	Correlation Co-Efficient
*Corynebacterium efficiens*	0.048	−0.319
*Bifidobacterium adolescentis*	0.014	−0.39
*Bifidobacterium pseudolongum*	0.025	0.357
*Alistipes unclassified*	0.033	0.342
*Desulfovibrio piger*	0.044	0.325

**Table 3:** Correlation coefficient and *p*-values of species associated with IFN-ɣ using PPDj month 3 post inoculation using Spearman rank correlation.

Species	*p*-Values	Correlation Co-Efficient
*Bacteroides thetaiotaomicron*	0.031	0.293
*Bacteroidales bacterium_ph*8	0.049	0.27
*Dorea formicigenerans*	0.012	0.34
*Faecalibacterium prausnitzii*	0.024	0.308
*Escherichia unclassified*	0.037	0.285

## Data Availability

All data generated or analysed during this study are included in this published article [and its supplementary informationsupplementary files].

## Supplementary Materials

The following supporting information can be downloaded at: https://www.mdpi.com/article/10.3390/ani13101652/s1, Table S1: LDA scores and p-vales associated for species in each group across time; Table S2: PERMANOVA and betadisper analysis for microbial community; Table S3: Superfocus level 1; Table S4: Level 3 Pathways; Table S5: A summary of the positive and inconclusive IDEX ELISA results during the course of the experimental challenge study, with S/P% provided. Animals which remained negative are not included. This table has been adapted from Britton, 2017; Table S6: IGRA PPDj ΔOD readings from animals at 3 months post inoculation; Figure S1: LEfSE analysis of Level 3 SUPER-FOCUS pathways in month 3; Figure S2: Bray-Curtis beta diversity among animals in both the exposed and control groups, across both time and herd. Month 6 shows the microbiome becoming more stable in herd B, where samples in the exposed group are most similar. However, results were non-significant.

## Abbreviations

DNA	Deoxyribonucleic acid
GIT	Gastrointestinal tract
IFN-ɣ	Interferon gamma
JD	Johne’s Disease
LDA	Linear discriminant analysis
MAP	*Mycobacterium avium* subsp. paratuberculosis
miRNA	microRNA
PERMANOVA	Permutational multivariate analysis of variance
qPCR	real-time PCR
