# Supplementary material for: Differences in Faecal Microbiome Taxonomy, Diversity and Functional Potential in a Bovine Cohort Experimentally Challenged with Mycobacterium avium subsp. paratuberculosis (MAP)
Source: Animals (Basel). 2023 May 16;13(10):1652. doi: 10.3390/ani13101652 (PMC10215331; doi:10.3390/ani13101652)
Supplement: Supplementary file 1 [file animals-13-01652-s001.zip › animals-2286853-supplementary.pdf]

Supplementary

Table S1. LDA scores and *p*-vales associated for species in each group across time.

Species	Time Point	Group	LDA	*p*-Value
Bifidobacterium pseudolongum	Month 3	Neg_Control	4.962739	0.011948
Methanobrevibacter unclassified	Month 3	Infected	4.886258	0.000113
Butyrivibrio unclassified	Month 3	Infected	4.777852	0.00039
Alistipes shahii	Month 3	Neg_Control	4.478157	3.15E-08
Parabacteroides distasonis	Month 3	Neg_Control	4.346065	0.00015
Bifidobacterium angulatum	Month 3	Infected	4.33015	0.002528
Prevotella stercorea	Month 3	Neg_Control	4.134405	0.000184
Bacteroides vulgatus	Month 3	Neg_Control	4.097004	0.015093
Subdoligranulum unclassified	Month 3	Neg_Control	4.093583	0.030568
Alistipes unclassified	Month 3	Neg_Control	4.076705	0.011057
Bifidobacterium adolescentis	Month 3	Infected	4.071279	0.000218
Bifidobacterium pseudolongum	Month 6	Neg_Control	5.08208	3.44E-07
Methanobrevibacter unclassified	Month 6	Infected	4.946584	0.025121
Butyrivibrio unclassified	Month 6	Infected	4.933534	0.005116
Butyrivibrio unclassified	Month 9	Infected	4.656766	0.046088
Lactobacillus buchneri	Month 9	Neg_Control	4.651226	0.016779

Table S2. PERMANOVA and betadisper analysis for microbial community.

**PERMANOVA**			**Betadisper**	
	**R^2**	***p*-value**		*p*-value
**Exposed vs. Control**	0.05509	0.001	**Exposed vs. Control**	0.00516
**Sero-positive vs. Sero-negative**	0.01083	0.05	**Sero-positive vs. sero-negative**	0.03714
**Month 3 vs. Month 6 vs. Month 9**	0.23819	0.001	**Month 3 vs. Month 6**	0.001
			**Month 3 vs. Month 9**	0.001
			**Month 6 vs. Month 9**	0.857
**Ileoceacal valve enlargement present vs. absent**	0.0065	0.207	**Ileoceacal valve enlargement present vs. absent**	0.34892

Table S3. Superfocus level 1.

Pathway Level 1	Time Point	Group	LDA	*p*-Value
**Membrane Transport**	Month 3	Neg_Control	3.333154	6.36E-06
**Cofactors, Vitamins, Prosthetic Groups, Pigments**	Month 3	Infected	2.821872	0.022913
**Phages, Prophages, Transposable elements Plasmids**	Month 3	Neg_Control	2.742754	0.000169
**Clustering based subsystems**	Month 3	Infected	2.695931	0.001186
**Potassium metabolism**	Month 3	Neg_Control	2.68032	7.54E-05
**RNA Metabolism**	Month 3	Infected	2.650863	0.019879
**Virulence**	Month 3	Neg_Control	2.649413	0.001114
**Stress Response**	Month 3	Infected	2.586105	0.00846
**Respiration**	Month 3	Infected	2.578904	0.025152
**Miscellaneous**	Month 3	Infected	2.57625	0.04115
**Predictions based on plant prokaryote comparative analysis**	Month 3	Infected	2.474448	0.015598
**Phages, Prophages Transposable elements**	Month 3	Neg_Control	2.418967	0.007609
**Iron acquisition and metabolism**	Month 3	Neg_Control	2.387186	0.022913
**Sulfur Metabolism**	Month 3	Infected	2.367102	0.046781
**Virulence, Disease and Defense**	Month 3	Neg_Control	2.356462	0.014847
**Regulation and Cell signaling**	Month 3	Neg_Control	2.241226	0.01278
**Carbohydrates**	Month 6	Neg_Control	4.724675	0.006332
**Protein Metabolism**	Month 6	Infected	4.65829	9.88E-05
**Cofactors, Vitamins, Prosthetic Groups, Pigments**	Month 6	Infected	4.338134	0.018525
**Nucleosides and Nucleotides**	Month 6	Neg_Control	4.284159	1.28E-05
**RNA Metabolism**	Month 6	Infected	4.268244	1.70E-05
**Respiration**	Month 6	Infected	4.109261	0.003931
**Fatty Acids Lipids and Isoprenoids**	Month 6	Neg_Control	3.756055	0.0202
**Phosphorus Metabolism**	Month 6	Neg_Control	3.667103	2.41E-07

**Phages, Prophages, Transposable elements, Plasmids**	Month 6	Infected	3.521126	0.001005
**Motility and Chemotaxis**	Month 6	Neg_Control	3.313241	3.19E-05
**Iron acquisition and metabolism**	Month 6	Neg_Control	3.312705	0.001713
**Nitrogen Metabolism**	Month 6	Neg_Control	3.312471	5.45E-08
**Virulence, Disease and Defense**	Month 6	Neg_Control	2.972411	0.000673
**Metabolism of Aromatic Compounds**	Month 6	Neg_Control	2.808811	0.015362
**Metabolism of Aromatic Compounds**	Month 9	Infected	2.012456	0.00512
**Phosphorus Metabolism**	Month 9	Neg_Control	2.30226	0.027486
**Nucleosides and Nucleotides**	Month 9	Neg_Control	2.405557	0.025121
**Cell Wall and Capsule**	Month 9	Neg_Control	2.457733	0.027486
**Motility and Chemotaxis**	Month 9	Neg_Control	2.67589	6.16E-05

Table S4. Level 3 Pathways.

**Pathway**	**Timepoint**	**Group**	**LDA**	***p* Value**
**Ton and Tol transport systems**	Month 3	Neg_Control	3.397796	6.37E-07
**Cellulosome**	Month 3	Neg_Control	2.852431	2.07E-06
**Conjugative transposon Bacteroidales**	Month 3	Neg_Control	2.765266	1.05E-05
**Mannose Metabolism**	Month 3	Neg_Control	2.725044	2.47E-06
**DNA replication**	Month 3	Neg_Control	2.496932	0.000711
**Multidrug Resistance Efflux Pumps**	Month 3	Neg_Control	2.462527	0.000109
**Peptidoglycan Biosynthesis**	Month 3	Neg_Control	2.442976	0.000258
**Translation elongation factor G family**	Month 3	Neg_Control	2.423262	2.81E-08

**Maltose and Maltodextrin Utilization**	Month 3	Infected	2.346961	0.027579
**Hydrogenases**	Month 3	Infected	2.319713	0.04115
**Aromatic amino acid interconversions with aryl acids**	Month 3	Infected	2.31549	0.000297
**ABC transporter oligopeptide TC3_A_1_5_1_**	Month 3	Infected	2.28926	0.018949
**Glycogen metabolism**	Month 3	Infected	2.279149	0.048796
**Glutamine, Glutamate, Aspartate and Asparagine Biosynthesis**	Month 3	Neg_Control	2.260514	0.008917
**RNA polymerase bacterial**	Month 3	Infected	2.24884	0.013439
**Universal GTPases**	Month 3	Infected	2.245888	0.00492
**Ribosome LSU bacterial**	Month 3	Infected	2.245667	0.025152
**Formate hydrogenase**	Month 3	Infected	2.241678	0.002063
**Potassium homeostasis**	Month 3	Neg_Control	2.224955	8.12E-05
**Histidine Degradation**	Month 3	Neg_Control	2.221785	0.02401
**DNA Repair Base Excision**	Month 3	Infected	2.215552	8.19E-06
**Glycolysis and Gluconeogenesis**	Month 3	Infected	2.214637	0.001263
**Branched Chain Amino Acid Biosynthesis**	Month 3	Neg_Control	2.206725	0.02401
**Ammonia assimilation**	Month 3	Neg_Control	2.189119	0.027579
**Single copy ribosomal proteins**	Month 3	Infected	2.187243	0.002191
**Fructooligosaccharides FOS and Raffinose Utilization**	Month 3	Infected	2.175969	0.014847

**Restriction Modification System**	Month 3	Neg_Control	2.173854	0.00492
**Competenceor DNAdamage inducible protein CinA and related protein families**	Month 3	Infected	2.168221	0.000169
**Cobalt, zinc, cadmium resistance**	Month 3	Neg_Control	2.147468	2.77E-05
**Multi drug Resistance, Tripartite Systems Found in Gram Negative Bacteria**	Month 3	Neg_Control	2.14152	3.64E-07
**tRNA mods Archaea**	Month 3	Infected	2.133358	0.003307
**DNA topoisomerases TypeII ATP dependent**	Month 3	Infected	2.130945	0.000195
**CBSS_350688_3_peg_1509**	Month 3	Infected	2.129998	0.030204
**DNA repair bacterial UvrDand related helicases**	Month 3	Infected	2.126481	3.99E-07
**Fatty Acid Biosynthesis FASII**	Month 3	Neg_Control	2.12425	0.013439
**Translation termination factors bacterial**	Month 3	Infected	2.122664	0.000277
**Respiratory dehydrogenases 1**	Month 3	Neg_Control	2.105421	0.000809
**Resistance to fluoroquinolones**	Month 3	Infected	2.102699	0.001114
**DNA repair bacterial**	Month 3	Infected	2.09602	0.001618
**DNA repair UvrABC system**	Month 3	Infected	2.092834	0.001522
**At5g37530_CsdL protein family**	Month 3	Infected	2.08672	0.002777

**Two cell division clusters relating to chromosome partitioning**	Month 3	Infected	2.070994	0.016383
**GroupII intron associated genes**	Month 3	Neg_Control	2.070567	0.000711
**At5g63290**	Month 3	Infected	2.069919	0.005201
**Heatshock dnaK gene cluster extended**	Month 3	Infected	2.036021	0.006835
**EC3_4_11__Aminopeptidases**	Month 3	Neg_Control	2.02731	0.001114
**Na translocating NADH quinone oxidoreductase and rnf_like group of electron transport complexes**	Month 9	Neg_Control	2.339701	0.001069
**Maltose and Maltodextrin Utilization**	Month 9	Neg_Control	2.28345	0.001636
**Glycogen metabolism**	Month 9	Neg_Control	2.236263	0.020915
**Carbon monoxide induced hydrogenase**	Month 9	Neg_Control	2.234515	0.003289
**Phosphate metabolism**	Month 9	Neg_Control	2.227462	0.004849
**Flagellum**	Month 9	Neg_Control	2.207519	2.89E-05
**Multidrug Resistance Efflux Pumps**	Month 9	Neg_Control	2.182279	0.015757
**Flagellar motility**	Month 9	Neg_Control	2.15775	1.26E-06
**CBSS_393121_3_peg_2760**	Month 9	Neg_Control	2.145124	0.001285
**Inositol catabolism**	Month 9	Infected	2.142067	0.000437
**DeNovo Purine Biosynthesis**	Month 9	Neg_Control	2.140344	0.001137
**Lactose and Galactose Uptake and Utilization**	Month 9	Neg_Control	2.121676	0.010635
**Restriction Modification System**	Month 9	Neg_Control	2.089932	0.015757
**Copper homeostasis**	Month 9	Infected	2.086014	0.000645
**Omega_amidaseKE2**	Month 9	Neg_Control	2.073458	0.000944
**Translation elongation factor G family**	Month 9	Neg_Control	2.067573	0.000208
**Lacto N BioseI and Galacto_N_Biose Metabolic Pathway**	Month 9	Neg_Control	2.041006	0.000467
**Methionine Biosynthesis**	Month 9	Infected	2.03467	0.000103
**Ni Fehydrogenase maturation**	Month 9	Infected	2.01025	0.003108
**Xylose utilization**	Month 9	Neg_Control	2.004691	0.032797

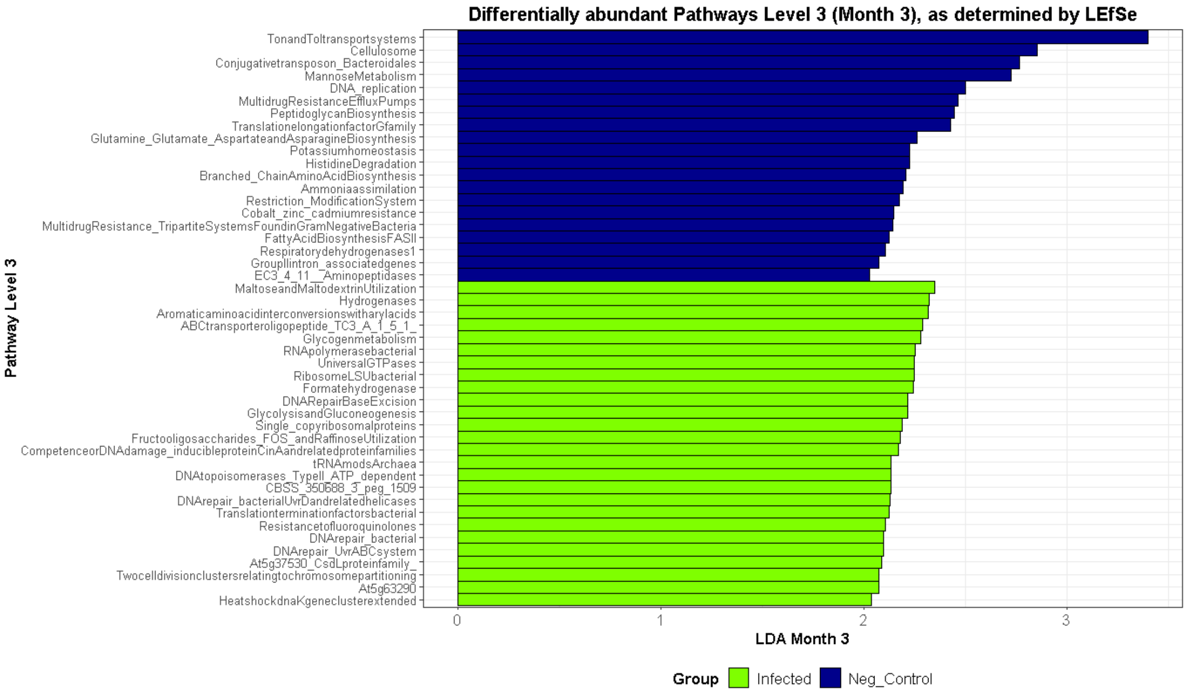

Figure S1. LEfSE analysis of Level 3 SUPER-FOCUS pathways in month 3.

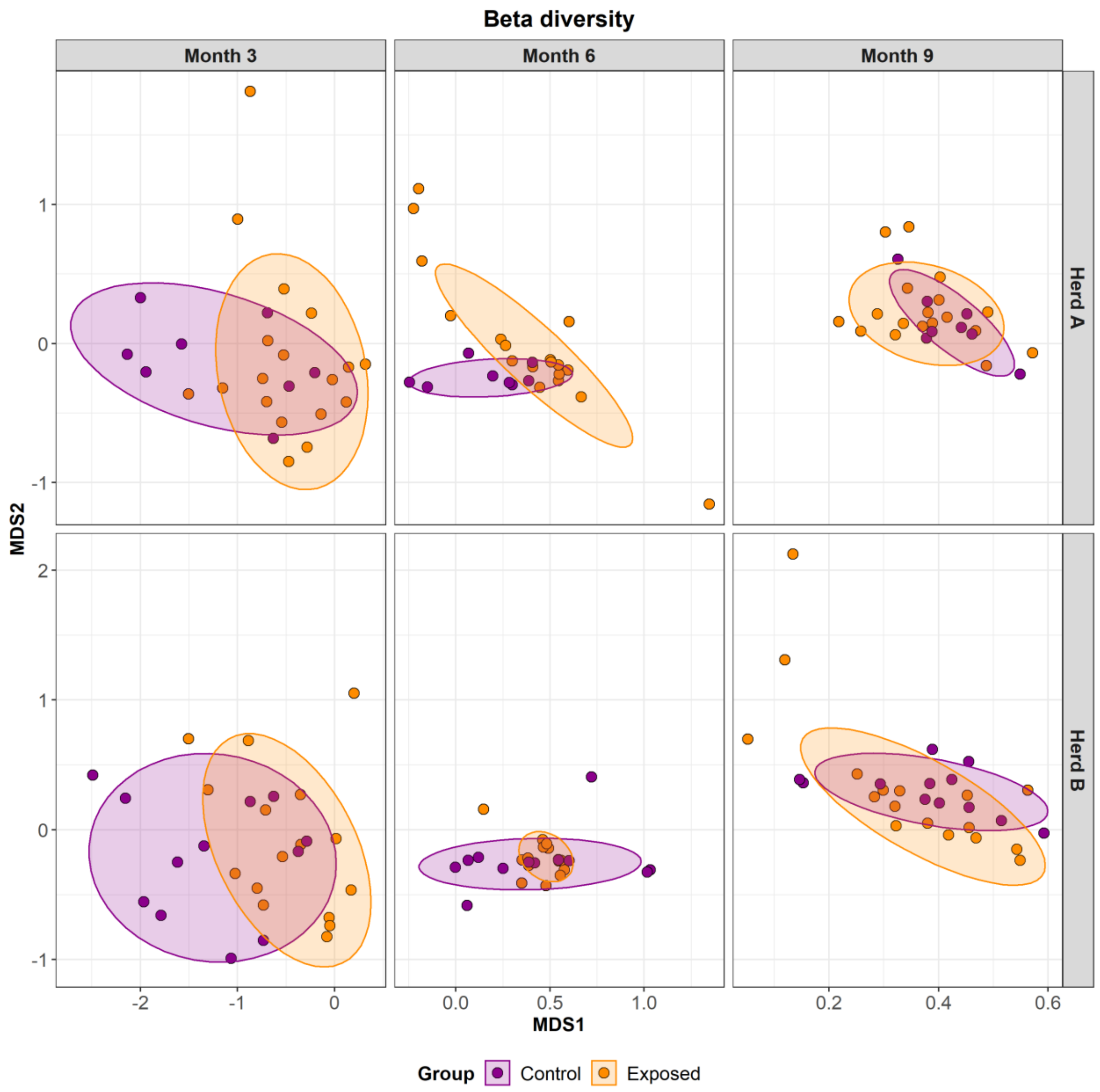

Figure S2. Bray-Curtis beta diversity among animals in both the exposed and control groups, across both time and herd. Month 6 shows the microbiome becoming more stable in herd B, where samples in the exposed group are most similar. However, results were non-significant.

Table S5. A summary of the positive and inconclusive IDEX ELISA results during the course of the experimental challenge study, with S/P% provided. Animals which remained negative are not included. This table has been adapted from Britton, 2017.

Group	ID	Pre	M3	M6	M10	M12	M15	M16	M20	M21	M24	M27	M28	M30	M31	M33
Challenged	2155	-	-	-	-	-	-	√ 68.3	√ 95.0	√ 55.6	√ 82.6	√ 86.9	√ 109.4	-	-	-
	2176	-	-	-	-	-	-	-	√ 56.8	-	I 52.5	-	-	-	-	-
	2194	-	-	-	-	-	-	I 50.2	-	I 51.4	√ 60.7	-	I 48.7	-	I 49.2	-
	2199	-	-	-	-	-	-	-	√ 61.6	I 50.2	-					
	2201	-	-	-	-	-	-	-	I 48.4	-	-	-	-	-	-	-
	2390	-	-	-	-	-	-	-	√ 63.0	√ 63.0	-	√ 64.8	√ 67.6	I 50.4	-	-
	2402	-	-	-	-	-	-	√ 93.9	√ 84.9	√ 68.7	-	√ 84.8	√ 99.0	√ 70.8	-	-
	2413	-	-	-	-	-	-	-	-	-	-	-	I 45.3	-	-	-
	2415	-	-	-	-	-	-	-	I 50.0	-	-	-	-	-	-	-
	2420	-	-	-	-	-	-	-	√ 68.1	√ 76.1	√ 70.8	√ 67.2	√ 85.2	√ 56.8	-	√ 81.8
Control	2170	-	-	-	√ 56.2	-										
	2454	-	-	-	-	-	-	-	-	-	√ 93.4	√ 78.3	√ 82.5	√ 68	√ 64.8	√ 79.8

Pre = premmune testing;M = month post-challenge; √ = Positive, - + Negative; I = Inconclusive. Blacked out cells represent animals that were necropsied during the previous timepoint.

Table S6. IGRA PPDj ΔOD readings from animals at 3 months post inoculation.

Group	ID	PPDj		ID	PPDj		ID	PPDj
Challenged	2149	0.369		2194	0.195		2403	0.012
	2152	2.947		2199	0.024		2404	0.315
	2154	1.817		2200	0.202		2407	0.161
	2155	0.005		2201	1.240		2408	0.439
	2158	0.248		2209	0.165		2410	0.116
	2162	0.115		2212	0.307		2412	0.193
	2164	0.087		2387	0.183		2413	0.051
	2169	0.141		2388	1.898		2415	0.148
	2176	0.222		2390	0.973		2416	0.204
	2182	0.512		2392	0.121		2420	0.411
	2183	0.072		2393	1.635		2422	0.140
	2185	0.061		2402	0.220			
	ID	PPDj		ID	PPDj		ID	PPDj
Control	2163	0.254		2218	0.011		2401	-0.010
	2168	2.636		2389	0.166		2405	0.681
	2170	0.022		2391	0.019		2406	0.398
	2172	0.148		2395	1.203		2409	0.062
	2175	0.324		2397	0.068		2425	0.191
	2213	1.377		2398	0.129		2454	0.771
	2215	0.212		2399	0.646			
